# Adapting *Drosophila melanogaster* Cell Lines to Serum-Free Culture Conditions

**DOI:** 10.1534/g3.120.401769

**Published:** 2020-12-01

**Authors:** Arthur Luhur, Daniel Mariyappa, Kristin M. Klueg, Kasun Buddika, Jason M. Tennessen, Andrew C. Zelhof

**Affiliations:** * Drosophila Genomics Resource Center, Indiana University, Bloomington, Indiana 47405; † Department of Biology, Indiana University, Bloomington, Indiana 47405

**Keywords:** *Drosophila*, cells, fly extract, serum-free, S2R+, Transcriptome, Metabolome

## Abstract

Successful *Drosophila* cell culture relies on media containing xenogenic components such as fetal bovine serum to support continuous cell proliferation. Here, we report a serum-free culture condition that supports the growth and proliferation of *Drosophila* S2R+ and Kc167 cell lines. Importantly, the gradual adaptation of S2R+ and Kc167 cells to a media lacking serum was supported by supplementing the media with adult *Drosophila* soluble extract, commonly known as fly extract. The utility of these adapted cells lines is largely unchanged. The adapted cells exhibited robust proliferative capacity and a transfection efficiency that was comparable to control cells cultured in serum-containing media. Transcriptomic data indicated that the S2R+ cells cultured with fly extract retain their hemocyte-specific transcriptome profile, and there were no global changes in the transcriptional output of cell signaling pathways. Our metabolome studies indicate that there were very limited metabolic changes. In fact, the cells were likely experiencing less oxidative stress when cultured in the serum-free media supplemented with fly extract. Overall, the *Drosophila* cell culture conditions reported here consequently provide researchers with an alternative and physiologically relevant resource to address cell biological research questions.

Pioneering research in the 1960s and 70’s led to the adaptation of *Drosophila melanogaster* cells in culture (Echalier and Ohanessian 1970; Schneider 1972). To successfully adapt primary cells from embryos, researchers designed media resembling the hemolymph of the third instar *Drosophila melanogaster* larva (Echalier 1997). Liquid media that successfully supported the establishment and continuous culture of *Drosophila* embryonic or imaginal disc cells include D22 medium (Echalier 1976), Schneider’s medium (Schneider 1972) and Shields and Sang’s M3 medium (Shields and Sang 1970; Cross and Sang 1978). The common features among these three media include higher osmotic pressure, increased amino acids, and divalent cation (Mg^2+^ and Ca^2+^) concentrations when compared to vertebrate cell culture media (Echalier 1997). Of these, the D22 medium recapitulates the larval hemolymph the best. However, it is also least well-defined as lactalbumin hydrolysate is used as the amino acid source, in addition to the use of the other undefined but common ingredient, yeast extract (YE) as a source of vitamins, nucleotides, trace metals and lipids (Echalier 1976; Echalier 1997). As compared to an earlier version of the Schneider’s medium, the one currently commercially available contains bacteriological peptone (BP) (Schneider 1964; Schneider 1972; Schneider and Blumenthal 1978). Nevertheless, the growth rate of S2 cells was unaffected when cultured in “reduced” Schneider’s media that did not contain BP (Sederoff and Clynes 1974), indicating that BP was not essential for S2 cell culture. In Shields and Sang M3 medium, lactalbumin hydrolysate was replaced by a defined content of amino acids (Shields and Sang 1970; Cross and Sang 1978). However, YE remained a component of the M3 medium as a source of vitamins. Subsequent successful cell line establishment from primary cultures have continued to rely on either Schneider’s or M3 media (Simcox *et al.* 1985; Ui *et al.* 1987; Niki *et al.* 2006; Simcox *et al.* 2008; Justiniano *et al.* 2012), underlining the continued utility of these *Drosophila* cell culture media formulations.

All three media compositions described above included fetal bovine serum (FBS), a key component used to support the cell culture. Prior to utilizing FBS, attempts to establish and culture *Drosophila* cells by supplementing the media with Lepidotera hemolymph proved to be unsuccessful (Schneider 1964). Hence, the understanding that a pool of mitogenic factors in FBS were key to establishing and supporting vertebrate cell and tissue cultures, led to the 5–20% supplementation of FBS to *Drosophila* cell cultures (Echalier 2018). Despite the success in establishing *Drosophila* cell cultures with FBS supplementation, batch variability and the toxicity of non-heat inactivated FBS prompted several attempts to eliminate or reduce FBS content to culture *Drosophila* cells (Echalier 1997).

One of the first attempts at *Drosophila* embryonic/imaginal disc primary cell culture in the absence of FBS and other non-physiological undefined components led to the development of ZW medium (Wyss 1982). ZW base medium supplemented with fly extract (FEx), bovine insulin and ecdysone created an environment for robust proliferation in addition to supporting survival and differentiation of embryonic/imaginal disc cells (Wyss 1982). FEx is a chemically undefined extract from whole flies, the preparation of which involves a 60° heat-treatment combined with ether extraction in earlier protocols (Wyss 1982; Currie *et al.* 1988; Echalier 2018). Wyss established that the active component(s) from FEx essential for supporting cell growth and proliferation is/are heat stable and resistant to ether extraction (Wyss 1982). FEx preparation that did not include ether extraction was sufficient to establish imaginal disc cell lines, albeit in the presence of reduced FBS (2%) serum supplementation (Currie *et al.* 1988). It has since been demonstrated that S2 cells can be adapted to media lacking either FBS or FEx, specifically as protein-synthesizing bioreactors (Galesi *et al.* 2007). However, multiple passaging of S2 cells was not supported, in variants of the Wyss’ ZO media lacking FEx (Burnette *et al.* 2014). Therefore, the options for culturing *Drosophila* cells in FBS-free culture conditions that support routine cell culture manipulations are extremely limited (Luhur *et al.* 2019b).

In this study we investigate whether S2R+ and Kc167 cells can be adapted to and maintained through several passages in serum-free culture conditions. We demonstrated that S2R+ and Kc167 cells can be grown in the absence of vertebrate serum by stepwise reduction in FBS with concomitant increase in FEx. The growth parameters of the FEx-adapted cells were comparable to cells grown in FBS. Freezing and thawing of FEx-adapted cells were not affected. Transfection efficiency, an important attribute for the utility of cell cultures, remained largely unchanged between FEx-adapted *vs.* FBS grown cells. Transcriptomic analyses of S2R+ cells cultured in FEx-supplemented media supported the earlier finding that S2R+ cells bear the signatures of cells from a hematopoietic origin. Lastly, metabolomic analyses of FEx adapted cells revealed largely comparable cellular metabolic content and reduced oxidative stress relative to FBS grown cells. Here, we present an alternative *Drosophila melanogaster* cell culture protocol that is more native and maintains the utility of the cell lines for routine cell culture procedures.

## Material and Methods

### Cell culture

**Figure 1:**
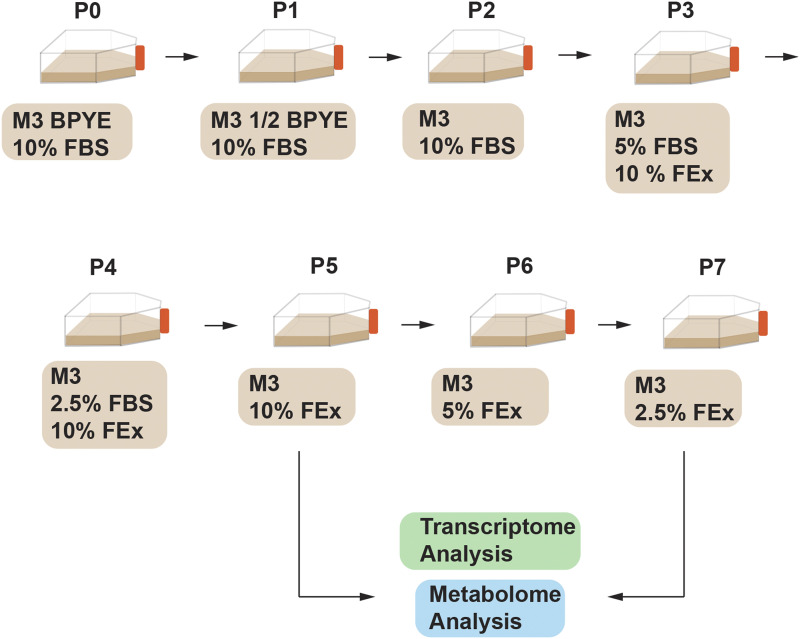
Adapting *Drosophila* S2R+ cells to FEx supplemented media. S2R+ cells were gradually adapted from M3 + BPYE + 10% FBS (MB10) into M3 + 10% FEx and M3 + 2.5% FEx. Transcriptomic and metabolomic analyses were carried out for control cells cultured in MB10 and adapted cells cultured in M3 + 10% FEx and M3 + 2.5% FEx.

Using best practices described (Luhur *et al.* 2019a; Luhur *et al.* 2019b) *Drosophila* S2R+ (DGRC Stock #150) cells were grown in Shield’s and Sang’s M3 insect media (Sigma) containing bactopeptone (BP) (Sigma), yeast extract (YE) (Sigma) and supplemented with 10% fetal bovine serum (FBS, Hyclone, GE Healthcare). The following procedure (Figure 1) was used to adapt S2R+ cultures to grow in M3 media supplemented with 10% fly extract (M3 +10% FEx). Cells were subcultured at a density of 2 × 10^6^ cells/mL in M3 +0.5X BPYE + 10% FBS for 1 passage, M3 +10% FBS for 1 passage, M3 + 5% FBS + 10% FEx for 1 passage, M3 + 2.5% FBS+ 10% FEx for 1 passage and finally in M3 + 10% FEx. Eventually, the cells were cultured in M3 + 5% FEx for 1 passage before being transferred to M3 + 2.5% FEx (Figure 1). Cells were subcultured every 2-3 days to maintain log-phase growth between 2 × 10^6^ cells/mL to 8 × 10^6^ cells/mL. For cryopreservation, cells cultured in serum containing media were frozen in M3 + BPYE + 20% FBS + 10% DMSO, while cells cultured in fly extract media were frozen in M3 + 20% FEx + 10% DMSO. To obtain the growth curve and doubling time, the cell density was measured at every 23-27 hr for a total of 98 hr.

Kc167 cells (DGRC Stock #1) were adapted from Hyclone-CCM3 media gradually to grow in 2.5% FEx- supplemented M3 or M3 + BPYE media. Briefly, Kc167 cells were transitioned 1:1 in CCM3:M3 mixed media in passage 1. The cells were then sub-cultured every 3-4 days to maintain log-phase growth between 2 × 10^6^ cells/mL to 8 × 10^6^ cells/mL for three more passages (using a 1:5 or 3:7 dilution in three different media conditions: M3 alone, M3 + 2.5% FEX, M3 + BPYE + 2.5% FEx), to progressively transition the cells to the new media conditions. Subsequent passages were maintained with a seeding density of 2 × 10^6^ cells/mL. For cryopreservation, all three adapted cell lines were frozen in M3 + 10% FEx + 10% DMSO. To obtain the growth curve and doubling time, the cell density was measured every 24 hr for a total of 98 hr.

All adapted cell lines: S2R+ FEx2.5% (DGRC Stock #310), S2R+ FEx10% (DGRC Stock #311), Kc167 M3 (DGRC Stock #312), Kc167 FEx2.5% (DGRC Stock #313), Kc167 MBFEx2.5% (DGRC Stock #314) are available at DGRC.

### Fly extract

Fly extract was derived from adult flies, males and females, (Oregon-R-modENCODE BL25211, Bloomington Drosophila Stock Center) within 1 week of eclosion. For a small-scale preparation, approximately 2 g of flies were needed to make 10 mL of extract. Briefly, 6.8 mL of ice-cold M3 media per gram of flies was used to homogenize the adult flies using a handheld blender (KitchenAid KHB1231ER) and then homogenized on ice by a motorized serrated Size C piston-type pestle (3431F25, Thomas Scientific) in a 55ml Size C Tissue Grinding Vessel (3431E55, Thomas Scientific). The homogenate was centrifuged at 1500 X g at 4° for 15 min. The supernatant was then incubated at 60° for 5 min, after which it is centrifuged at 1500 X g at 4° for 90 min. The resulting supernatant was sterile filtered (0.2 μm) to constitute the fly extract that can be supplemented to culture media or stored at -20°.

### Statistical analysis

To analyze the cell population doubling time, non-linear curve fit was carried out for the various growth profiles using an exponential growth equation (Prism 7.0). We modeled the growth curve with the exponential growth equation using least squares regression method. We asked if the best-fit value of the rate constant (K) of the unshared parameter differ between the data sets. Both the Extra sum-of-squares F test and the Akaike’s Informative Criteria (AICc) were separately used to compare the best-fit values between the data sets. In all comparison methods, the parameter tested was rate constant (K), from which the doubling time can be derived (Doubling time = ln(2)/K). Welch ANOVA multiple comparison test with Tukey correction was used to determine the statistical significance of the mean GFP and transfection intensity of the cells.

### Transfection and fluorescent-activated cell sorting

Cells were transfected with ptubGAL4 (Lee and Luo 2001) and pUAS-GFP plasmids. Briefly, 3 X 10^6^ cells (2 mL at 1.5 X 10^6^ cells/mL) were transfected in 6-well plates. 600 ng of ptub-GAL4 and 600 ng of pUAS-GFP plasmids were mixed 100 μL of buffer EC, 16 μl of Enhancer solution and 16 μl of Effectene reagent (Qiagen). The transfection efficiency was quantitated at 96 hr after transfection using fluorescent-activated cell sorting on a FACS Aria II (IUB Flow Cytometry Core Facility), comparing the population of live GFP (positive) cells to the total live cell population (DAPI negative cells).

### Microscopy and immunofluorescence

Live cells were imaged using Nikon Eclipse TS100 brightfield microscope on a 40X objective. For fluorescent imaging, cells were fixed using 4% paraformaldehyde/1X PBS for 10 min. The cells were then washed in 1X PBS and subsequently mounted on mounting media containing DAPI (Vectashield H-1200) before being imaged on a Leica SP8 Confocal Microscope.

### Total RNA isolation

Total RNA was isolated from S2R+ cells (5 X 10^6^ cells) harvested from the mid-log phase of growth using Trizol LS reagent (Invitrogen) according to the manufacturer’s protocol. Total RNA was subsequently cleaned using RNEasy Mini Kit (Qiagen) according to the manufacturer’s instructions.

### Cell transcriptome analysis

Demultiplexing of the reads was performed with bcl2fastq version 2.20.0 pipeline. Reads were mapped to the *Drosophila melanogaster* dmel_r6.30 using Hisat2 (version 2.1.0). Mapped reads were counted using Samtools (version 1.9) and featureCounts (version 1.6.4). The input files and raw output files have been deposited in NCBI Gene Expression Omnibus. Read normalization for transcriptome analysis was performed using the R Bioconductor package edgeR (https://bioconductor.org/packages/release/bioc/html/edgeR.html) (Robinson *et al.*, 2010). The read counts were normalized using Trimmed Mean of M-values method (TMM) from edgeR to compare between samples (Robinson *et al.* 2010). The R Bioconductor package DESeq2 was used for identifying differentially expressed genes (https://bioconductor.org/packages/release/bioc/html/DESeq2.html).

Data visualization steps were done as described using custom scripts written using R (Buddika *et al.* 2020). Data visualization R scripts in Figure 3 are available upon request. Significantly up- or down-regulated genes were used to identify enriched Gene Ontology (GO) terms using the g:Profiler overrepresentation analysis (ORA) function g:GOSt (https://biit.cs.ut.ee/gprofiler/gost). A selected significantly enriched biological processes GO categories were plotted using R.

### Cell metabolome analysis

For all conditions, cells were plated at a cell count of 1 × 10^6^ and harvested at mid-log phase (4-5 × 10^6^ cells/mL). Either control S2R+ cells cultured in M3 + 10% FBS or S2R+ cells pre-adapted to 2.5% FEx culture condition grown in M3 + 2.5% FEx were pelleted. The respective culture supernatants and pellets were snap frozen in liquid nitrogen and stored in -80° (n = 6). In parallel, both M3 + 10% FBS and M3 + 2.5% FEx media that were not seeded with cells were incubated under identical conditions, immediately frozen in liquid nitrogen and stored in -80°. All the frozen samples were submitted to Utah Metabolomics Core for metabolite extraction and GC-MS based targeted metabolomic analysis.

For extraction, cold 90% methanol (MeOH) solution was added to each sample to give a final concentration of 80% MeOH to the cell pellet. Samples were then incubated at -20° for 1 hr. After incubation the samples were centrifuged at 20,000 × g for 10 min at 4°. The supernatant was then transferred from each sample tube into a labeled, fresh micro centrifuge tube. Pooled quality control samples were made by removing a fraction of collected supernatant from each sample and process blanks were made using only extraction solvent and no cell culture. The samples were then dried *en vacuo*.

### Mass Spectrometry and data analysis of samples

All GC-MS analysis was performed with an Agilent 5977b GC-MS MSD-HES and an Agilent 7693A automatic liquid sampler. Dried samples were suspended in 40 µL of a 40 mg/mL O-methoxylamine hydrochloride (MOX) (MP Bio #155405) in dry pyridine (EMD Millipore #PX2012-7) and incubated for one hour at 37° in a sand bath. 25 µL of this solution was added to auto sampler vials. 60 µL of N-methyl-N-trimethylsilyltrifluoracetamide (MSTFA with 1% TMCS, Thermo Fisher Scientific #TS48913) was added automatically via the auto sampler and incubated for 30 min at 37°. After incubation, samples were vortexed and 1 µL of the prepared sample was injected into the gas chromatograph inlet in the split mode with the inlet temperature held at 250°. A 10:1 split ratio was used for analysis for the majority of metabolites. Any metabolites that saturated the instrument at the 10:1 split were analyzed at a 200:1 split ratio. The gas chromatograph had an initial temperature of 60° for one minute followed by a 10°/min ramp to 325° and a hold time of 10 min. A 30-meter Agilent Zorbax DB-5MS with 10-meter Duraguard capillary column was employed for chromatographic separation. Helium was used as the carrier gas at a rate of 1 mL/min.

Data were collected using MassHunter software (Agilent). Metabolites were identified and their peak area was recorded using MassHunter Quant. Metabolite identity was established using a combination of an in-house metabolite library developed using pure purchased standards, the NIST library and the Fiehn library. Data were analyzed using the “MetaboAnalyst” software tool. For the pathway analysis, all the metabolites that were significantly enriched in a particular set of comparisons were assessed using the MetaboAnalyst pathway analysis module (Xia and Wishart 2010). The parameters used for the pathway analysis module include the *Drosophila melanogaster* pathway library, Fisher’s Exact test to determine the over representation analysis and the relative betweenness centrality as the node importance measure to determine the pathway topology.

### Data availability

All data necessary for confirming the conclusions in this paper are included in this article and in supplemental figures and tables. All cell lines are available upon request from the DGRC, all metabolomic data are included in the manuscript and transcriptomic data has been deposited NCBI Gene Expression Omnibus – accession GSE154485. Supplemental material available at figshare: https://doi.org/10.25387/g3.13050818.

## Results and Discussion

### *Drosophila* cell lines, S2R+ and Kc167, can be adapted to grow in the absence of fetal bovine serum

The embryonically derived *Drosophila* cell line S2R+ cells are grown in Shields and Sang M3 medium, supplemented with bactopeptone and yeast extract (BP+YE) and 10% FBS (abbreviated as MB10). Here, we report the effects of culturing S2R+ line in M3 media lacking BPYE and FBS, instead gradually replacing these components with varying concentrations of *Drosophila* fly extract (FEx). Briefly, S2R+ or Kc167 cells were progressively adapted to grow in a media lacking FBS. Due to widespread cell death upon immediate transition into FBS free culture FEx supplemented media, it was imperative that the transition was gradual. Concomitantly, adult *Drosophila* FEx was supplemented at 10% (v/v) and eventually reduced to 2.5% (v/v) (Figure 1). The additional BP and YE were also excluded from the final media formulation.

**Figure 2:**
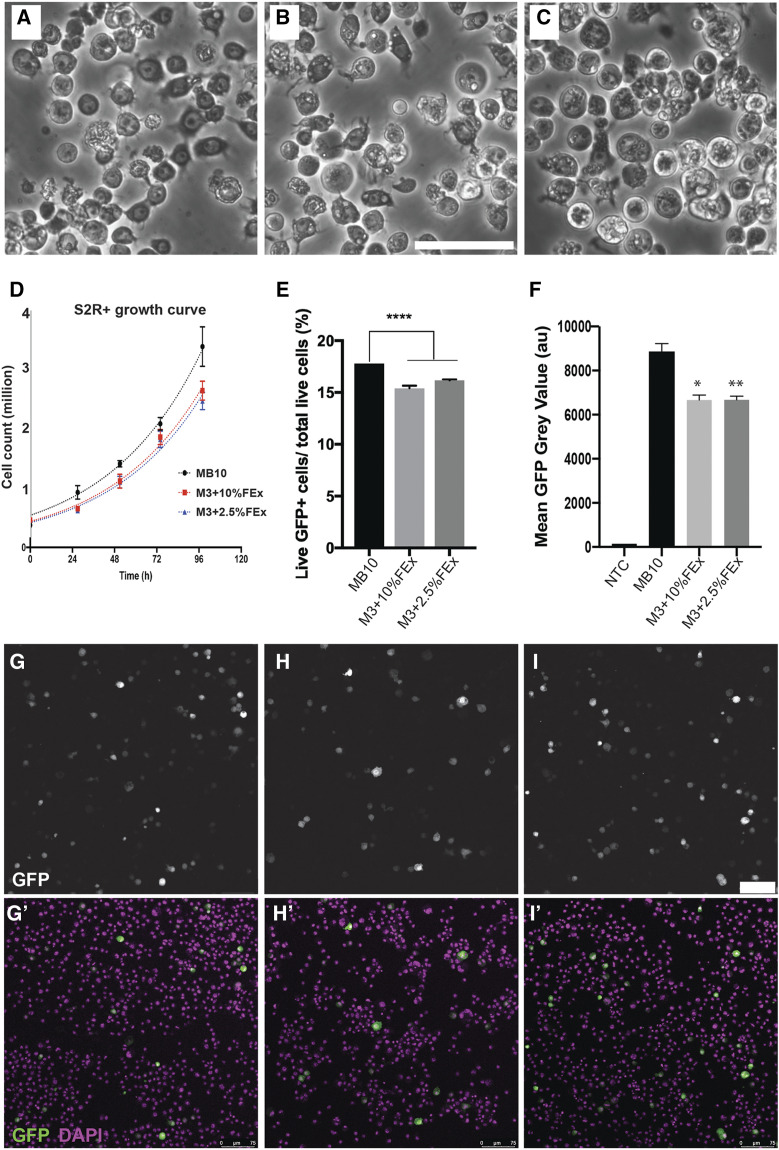
Properties of S2R+ cultures across three culture conditions. (A) The morphology of S2R+ cells grown in M3 + BPYE + 10% FBS (Control). (B) The morphology of S2R+ cells in M3 + 10% FEx. (C) The morphology of S2R+ cells in M3 + 2.5% FEx. Scale bar, 50 μm. (D) The growth curves of S2R+ grown in three distinct media as indicated. (E) A bar graph quantitating the transfection efficiency of S2R+ cells grown in three distinct media as determined by flow cytometry. (n = 3, **** denotes *P* < 0.0001). (F) A bar graph quantitating the GFP expression intensity of S2R+ cells grown in the three distict media as determined by flow cytometry. (n = 3, * denotes *P* = 0.0416 and ** denotes 0.0085, relative to MB10 control), NTC – non-transfected control. (G) Micrograph of S2R+ cells transfected with ptub-GAL4 and pUAS-GFP grown in control media, (H) M3 + 10% FEx and (I) M3 + 2.5% FEx. Scale bar, 75 μm. GFP expression labels cells harboring ptub-GAL4 and pUAS-GFP. DAPI marked the total cells.

We then examined three general properties relevant to the utility of these lines for research application: morphology, growth profiles and transfection efficiency. S2R+ cells stably cultured in M3 + Fly extract (M3 + FEx) media for at least 10 passages shared indistinguishable cellular morphology (Figure 2A-C). The proliferative capacities of the cells were not significantly altered as quantified by the population doubling time (*T_d_*). The *T_d_* of S2R+ cells cultured in MB10, M3 + 10% FEx, and M3 + 2.5% FEx are in the range of 31.87 - 39.93 h, 34.36 - 42.15 h and 35.23 - 44.61 h, respectively (Figure 2D). AICc and F test of the non-linear fitting of the growth curves indicate that the doubling times were not significantly different (*P* = 0.1833 and *P* = 0.3385, respectively). We next measured the transfection efficiency of these cells by measuring the GFP expression resulting from co-transfection of ptub-GAL4 and pUAS-GFP plasmids. Only cells that harbor both plasmids will express cytoplasmic GFP. Overall, S2R+ cells displayed a small but statistically significant reduction in transfection efficiency when cultured in 10% FEx and 2.5% FEx containing media (15.2% and 16.1% respectively), compared to control cells (17.8%) (Figure 2E-I). The mean transgene expression strength was also reduced by 25% in cells cultured in M3 + 10% FEx (*P* = 0.04) and in M3 + 2.5% FEx (*P* = 0.0085), when compared to cell cultured in MB10 (Figure 2F-I). This culture condition could therefore provide an alternative for *Drosophila* cell cultures models that have cellular toxicity associated with the high levels of transgene over-expression (Cherbas and Gong 2014).

With the successful adaptation of S2R+ cells, we also tested whether we could adapt another commonly used embryonic derived cell line, Kc167, to medium with or without fly extract, potentially revealing a media recipe that would be beneficial for many different *Drosophila* cell lines. Kc167 cells have traditionally been cultured in the proprietary HyClone-CCM3 serum-free media, in which the ingredients are completely unknown, or in M3 + BPYE + 10% FBS. For our comparisons we utilized the following media conditions: CCM3, M3 media alone, M3 + BYPE + 2.5% FEx, and M3 + 2.5% FEx. In all conditions, we observed that Kc167 cells had identical morphology (Figure S1A-B), but cells in M3 media alone were more prone to form aggregates during log phase. The cells proliferated slower in both M3 (*T_d_* = 52.5 – 89 h) and M3 + 2.5% FEx (*T_d_* = 54.4 - 80.23h), compared to control cells in CCM3 media (*T_d_* = 47.72 - 77.4h) (Figure S1C). Whereas, cells cultured in M3 + BPYE + 2.5% FEx displayed comparable growth profile (*T_d_* = 47.26h – 67.4h) as cells in CCM3 media (Figure S1C). Moreover, the media conditions did not prevent the ability to transfect the cells but there were notable differences in both the strength of transgene expression and transfection efficiency (Figure S1D-E). Generally, Kc167 cells cultured in M3 alone or in M3 + 2.5% FEx exhibited higher transfection efficiency (47.4% and 40.4%, respectively), compared to Kc167 cells cultured in CCM3 (29.1%). Notably, supplementing BPYE to M3 + 2.5% FEx resulted in the lowest transgene expression and transfection efficiency (Figure S1D-E). Our data demonstrated that other than the slightly slower growth rates, Kc167 cells were fully adaptable to our M3 + 2.5% FEx media. Lastly, for the importance to the utility of these adapted lines, all cell lines, S2R+ and Kc167, could be cryopreserved in a serum-free media and subsequently revived. Overall, in terms of the general utility of the S2R+ and Kc167 cells adapted to fly extract, we believe none of the noted different parameters hamper the utility of these cells for transgene analyses. As such, we have designated these FEx-adapted cell lines as new resources available for further biological experimentation.

### The transcriptome of cells cultured in FEx retain a hemocyte transcriptional profile

**Figure 3:**
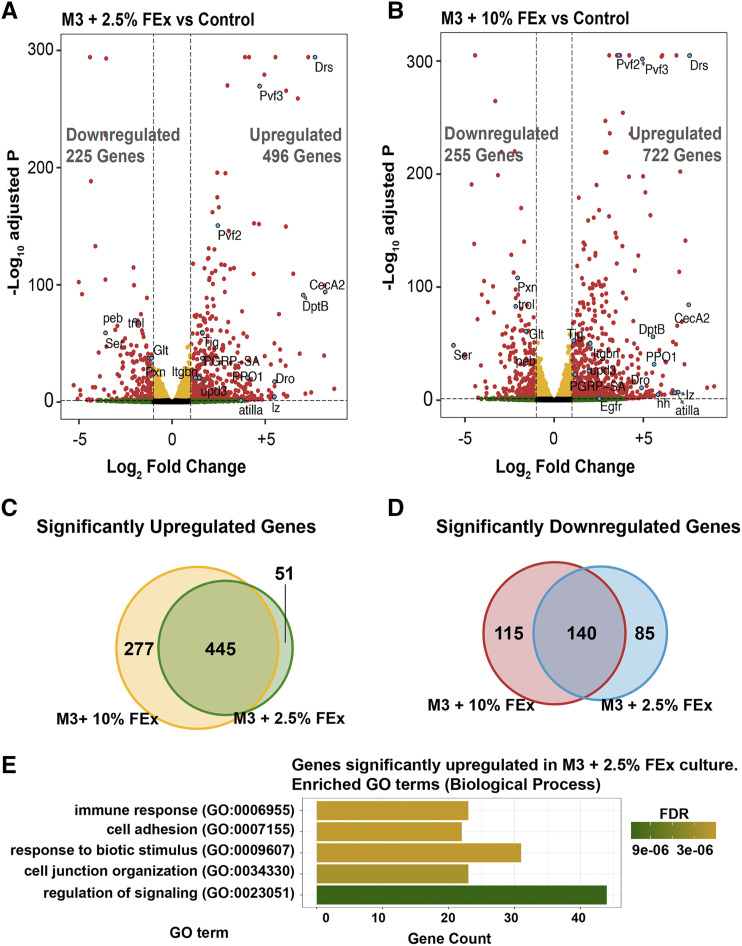
Differential gene expression in S2R+ cultures across three culture conditions. (A) Volcano plot of differentially expressed genes from comparison of cells cultured in M3 + 2.5% FEx to control or (B) M3 + 10% FEx to control. Each dot represents a single gene. Yellow indicates a false discovery rate adjusted p-value (FDR) < 0.05 and a Log2 fold change < 1 or > -1. Green indicates Log_2_ fold change > 1 or < -1 and an FDR >= 0.05. Red indicates an FDR < 0.05 and Log_2_ fold change > 1 or < -1. A select number of genes highlighted in cyan were discussed in the text. Venn diagram showing the overlap of (C) significantly upregulated (FDR < 0.05 and Log_2_ fold change > 1) or (D) significantly downregulated (FDR < 0.05 and Log_2_ fold change < -1) genes in M3 + 2.5% FEx and M3 + 10% FEx compared to control cultures. (E) Bar plot showing a selected set of significantly enriched Gene Ontology (GO) terms for genes that are significantly upregulated (adjusted *P* < 0.05 and log_2_ fold change > 1) in cells cultured in M3 + 2.5% FEx compared to control.

S2R+ cell line is one of the most commonly used *Drosophila* cell lines and, as such, we chose this line to investigate whether the change in culture conditions was drastically altering the nature of these cells. S2R+ cells were derived from the late embryonic stages of development (Schneider 1972) and these cells exhibited a transcriptional profile indicative of cells of the hematopoietic origin (Cherbas *et al.* 2011). To investigate the nature of these cells, we used RNA-seq to survey and compare the transcriptomes of S2R+ lines cultured in MB10, M3 + 10% FEx, and M3 + 2.5% FEx (Figure S2A). Any transcript with a normalized expression value greater than 10 in each replicate was considered to be expressed in S2R+ cells under each culture condition. We identified 6549 expressed genes in the three culture conditions (TMM normalized expression value > 10) and 94% (6164/6549) of the genes were commonly expressed in all conditions (Figure S2B). 110, 28 and 50 genes were exclusively expressed in cells cultured in MB10, M3 + 2.5% FEx and M3 + 10% FEx, respectively (Figure S2B). To query the overall similarity of the three datasets, we calculated Spearman and Pearson correlation coefficients for each pairwise comparison. Our results indicated a strong positive correlation between transcriptomes, indicating that the overall gene expression profiles for the three conditions are globally similar (Figure S2C). Despite this global similarity, we identified 721 and 977 differentially expressed (DE) transcripts (false discovery rate adjusted p-value (FDR) <0.05 and Log_2_ fold change > 1 or < -1) in cells cultured in M3 + 2.5% FEx and M3 + 10% FEx, respectively when compared to the control cells in MB10 (Figure 3A-B). Notably, 445 common transcripts were upregulated in cells cultured in the two different FEx supplemented media (Figure 3C). Similarly, 140 transcripts were commonly down-regulated (Figure 3D). Gene ontology (GO) enrichment analysis of the upregulated genes in cell cultured in M3 + 2.5% FEx revealed multiple categories that fall broadly into immune response, cell adhesion, cell junction organization and cell signaling (Figure 3E). In contrast, similar analysis for the transcripts that were downregulated did not significantly enrich for any biological processes GO terms.

The GO terms enriched for genes upregulated in S2R+ FEx adapted cells were not surprising given the origin of cell line and the culture conditions. The high expression of genes involved in mounting an immune response could be attributed to the cells responding to factors present in the fly extract. Such factors may include the soluble bacterial lipopolysaccharide (LPS) and/or insoluble peptidoglycans that may be present in the extract. These factors can induce the upregulation a number of innate immune response genes including *PGRPs*, *Diptericins*, *Cecropins*, *Attacins* and *Metchnikowin*, as has been reported in the *Drosophila* mbn-2 cell line (Choe *et al.* 2002).

Previous tiling array and RNA-seq experiments have suggested that S2 lines to be of hematopoietic origin (Cherbas *et al.* 2011) and these FEx-adapted S2R+ lines also maintain the expression of many of classic hematopoietic markers. Cells cultured in FEx supplemented media had elevated transcripts for the ligands (*pvf2* and *pvf3*) of the PDGF/VEGF (Platelet-Derived Growth Factor/Vascular Endothelial Growth Factor)-receptor related (PVR) signaling pathway (Clauss 2000) (Figure 3A-B, Table S1, S2). The PVR pathway has been shown to be necessary for hemocyte viability in cultured cells and *in vivo* (Brückner *et al.* 2004). There are three types of mature hemocytes in *Drosophila*: phagocytic plasmatocytes, crystal cells with roles in innate immunity, and lamellocytes, which respond to parasitic wasp infections (Banerjee *et al.* 2019). Our data also indicated reduced transcript expression for plasmatocytes markers: *Peroxidasin* (*pxn*) (Kurucz *et al.* 2007) and trol the *Drosophila* ortholog of mammalian Perlecan (Matsubayashi *et al.* 2017), but an increase in integrins (*itgbn*) important for phagocytosis (Nonaka *et al.* 2013) (Figure 3A, 3B, Table S1, S2). The expression of crystal cells markers *Prophenoloxidase* *1* (*ppo1*) (Binggeli *et al.* 2014; Neyen *et al.* 2015) and *lozenge** (**lz*) (Lebestky *et al.* 2000) were also increased. In addition, we noted an increased expression of lamellocyte markers, such as *atilla* (Kurucz *et al.* 2007) and α-PS4 integrins (Irving *et al.* 2005) (Figure 3A, 3B, Table S1, S2). Circulating hemocytes also secrete extracellular matrix (ECM) proteins such as Tiggrin (*tig*) (Fogerty *et al.* 1994), Glutactin (*glt*) (Fessler and Fessler 1989) and Lamins (Kusche-Gullberg *et al.* 1992; Banerjee *et al.* 2019). Notably, S2R+ cells cultured in media supplemented with two distinct fly extract concentrations exhibited changes in the expression of transcripts encoding for these molecules (Figure 3A, 3B, Table S1, S2).

Withdrawing FBS and introducing the exogenous fly extract into the culture media may alter the regulation of signaling pathways (Figure 3E). From the RNAseq data, we queried the transcriptional output for the ligands and the corresponding receptors for the major signaling pathways including Insulin, Notch, JAK/STAT, EGFR, Hippo, TGF-b, Hedgehog, TNF-a, TOR and Wnt pathways. We found that the transcripts for factors in the JAK/STAT pathway, which are known to regulate hemocyte proliferation: *upd2* (2.4 fold) and *upd3* (2.9 fold), *dome* (1.5 fold) and *socs36E* (2.5 fold) were elevated (Figure 3A, B, Table S1, S2). This result paralleled the observation that *upd3* transcripts were elevated in adult hemocytes as an immune response to septic injury (Agaisse *et al.* 2003; Chakrabarti *et al.* 2016). The Notch signaling pathway is known to regulate hemocyte differentiation (Duvic *et al.* 2002; Lebestky *et al.* 2003; Banerjee *et al.* 2019) and we noted that the transcript expression for the Notch ligand, Serrate (*Ser*) was strongly dampened (11.8 fold) (Figure 3A, B Table S1, S2) in cells cultured in the FEx-supplemented media, suggesting an insight into the cellular differentiation status. The transcript expression for members of the other signaling pathways did not appear to be substantially altered in cells cultured in M3 + 2.5% FEx (Table S1 and S2). While we did not observe any clear changes in the general properties of the cells cultured in M3 + 2.5% FEx compared to the cells cultured in M3 + 10% FEx, the numbers of differentially expressed genes were higher when the cells were cultured in M3 + 10% FEx media than in M3 + 2.5% FEx media. In addition, the relative strength on differential gene expression levels of transcripts in the signaling pathways, for instance *Egfr*, *hh*, *upd3*, *pvf2* and *Ser* were generally more pronounced in cells cultured in 10% FEx than it was in 2.5% FEx (Figure 3A, 3B, Table S1-S3). In fact, the transcript for hedgehog (*hh**)*, was elevated in cells cultured in M3 + 10% FEx, and not in cells cultured in M3 + 2.5% FEx (Figure 3B and Table S2). It is important to note that transcriptional changes may not reflect the output of the signaling pathways. Overall, how the described transcriptomic changes impact cellular function of the S2R+ cells are not known. Nonetheless, the transcriptomic data do not indicate a dramatic change in transcription upon the switch to FEx and considering the general properties for the utility of the cells and their transcriptome, we recommend that S2R+ cells to be cultured in M3 + 2.5% FEx.

### Culturing S2R+ cells in fly extract supplemented media did not alter secreted cellular metabolites

**Figure 4:**
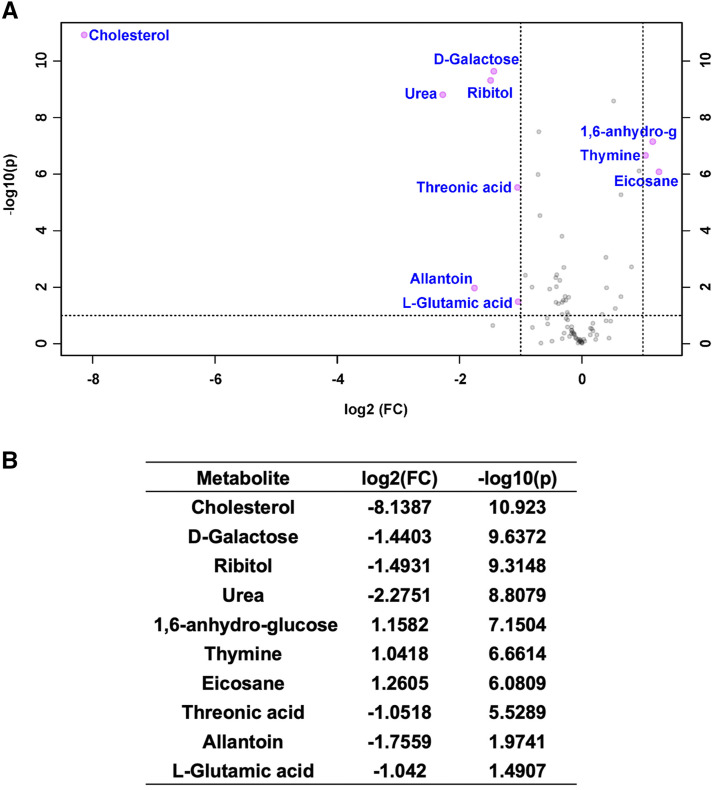
The cellular metabolomes of S2R+ cells grown in FBS- or FEx-supplemented media are comparable. (A) A volcano plot of all the metabolites assessed. Metabolites that are significantly (*P* < 0.05) changed when the S2R+ cells were grown in M3 + 2.5% FEx- *vs.* FBS-supplemented media by at least twofold are highlighted in pink. (B) List of metabolites that significantly (*P* < 0.05) change by at least twofold in cells grown in FEx- *vs.* FBS-supplemented media are listed.

Adapting cells to new conditions may alter metabolic pathways. We investigated the extent of changes to the a) targeted metabolome and b) secreted metabolites of cells cultured in FBS or M3 + 2.5% FEx adapted cells. Cell pellets, culture supernatants, and the cell-free media subjected to identical conditions were analyzed using a targeted metabolomics approach designed to measure the relative abundance of small polar molecules. A Principal Component Analysis of the FBS- *vs.* FEx-cultured cellular metabolomic profiles revealed that they were significantly different (Figure S3). However, only 10 out of 119 (8.4%) metabolites assessed were significantly different between the FBS- *vs.* FEx-cultured cellular metabolomes (Figure 4A, B). Interestingly, among the ten metabolites that were significantly changed by at least twofold (*P* < 0.05) in FEx- *vs.* FBS-cultured cells, the levels of six of them (cholesterol, urea, ribitol, 1,6-anhydro glucose, threonic acid and allantoin) were also significantly changed in a similar direction when comparing cell-free FEx *vs.* FBS media (Figure 4A, B, S4A, B). Considering that *Drosophila* metabolism is unable to synthesize cholesterol, ribitol, 1,6-anhydro glucose, or threonic acid (Clark and Block 1959; Thomas and Hughes 1983; Simoneit *et al.* 1999) and allantoin/urea metabolic pathway differs significantly between insects and mammals (Hinton 1956; Wallrath *et al.* 1990; Samson 2000), the differences observed in these six metabolites can be simply attributed to the FEx or FBS-supplementation of the base M3 medium. Only 4/19 of mostly nucleobase metabolites (adenine, thymine, uracil and lauric acid) were uniquely changed between culture supernatants from FBS- or FEx-supplemented cell cultures as compared to similar cell-free conditions (Fig S4 C, D). Therefore, our analysis suggests that the steady state concentrations of key intermediary metabolites was similar when S2R+ cells were cultured in either FBS- or FEx-supplemented media.

**Figure 5:**
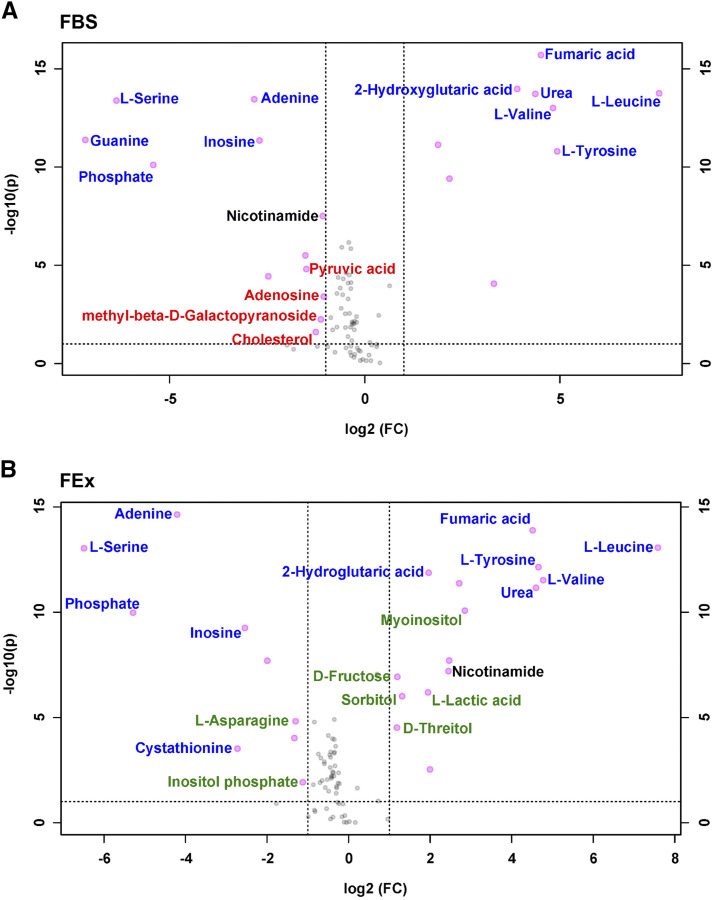
Metabolic output of S2R+ cells grown in FBS- or FEx-supplemented media are similar. (A) Volcano plot of metabolites comparing FBS-supplemented cell-free media *vs.* FBS-supplemented culture supernatant. The metabolites that are uniquely changing in FBS-supplemented culture supernatant are labeled in red (B) Volcano plot of metabolites comparing cell-free media *vs.* 2.5% FEx-supplemented culture supernatant. The metabolites that are uniquely changing in FEx-supplemented culture supernatant are labeled in green. Metabolites that are significantly (*P* < 0.05) changed when the S2R+ cells were grown in cell-free media *vs.* the respective FEx- or FBS-culture supernatant by at least twofold are highlighted in pink. Metabolites that are pink but unlabeled or labeled in blue in either panels change similarly irrespective of the media being supplemented with FBS or FEx. Nicotinamide, the only metabolite to display opposing changes depending on whether the cells were cultured in FEx or FBS is labeled in black.

To determine the changes in the secreted metabolites from cells cultured in either FEx- or FBS-supplemented media, the targeted metabolomes of the culture supernatants and their respective cell-free media were compared. Principal Component Analyses of both these comparisons determined that the metabolomic profile in either case was significantly different between the respective culture supernatants as compared to cell-free media (Figure S5 A, B). The number of metabolites with significantly (*P* < 0.05) different levels in the appropriate cell-free media *vs.* FBS- and FEx-supplemented culture supernatants, were 30 and 25 metabolites, respectively (Figure 5A, B). Nevertheless, 57% (17/30) and 68% (17/25) of the metabolites that changed were common between both the analyses (Table S4). We observed an increase in lactic acid levels secreted by cells grown in FEx-supplemented media, an observation that parallels the switch to aerobic glycolysis in *Drosophila* macrophages, an indicator of S2R+ cells favoring a metabolic profile reflective of their hematopoietic origin (Krejčová *et al.* 2019) (Figure 5B). Contrasting changes were observed in nicotinamide levels with significant increase of the metabolite in FEX-supplemented culture supernatant and a concomitant reduction in FBS-supplemented culture supernatant (Figure 5, Table S4). This depletion of nicotinamide upon culturing cells in FBS-supplemented media was despite the cell-free FBS-supplemented media having a significantly higher level of nicotinamide as compared to FEx-supplementation (Fig. S4B). In *Drosophila*, nicotinamide is a precursor to synthesize NAD – an essential cofactor involved in cellular redox metabolism (Rongvaux *et al.* 2003). This increased requirement for nicotinamide in FBS-supplemented cell culture could indicate that cells growing under these conditions might need to contend with elevated oxidative stress. To establish if any particular metabolic pathway is over-represented among the metabolites that are significantly changed in the supernatants on S2R+ culture, we used the MetaboAnalyst pathway analysis module to analyze data from FBS- or FEx-supplemented media. Similar metabolic pathways were over-represented significantly on S2R+ cell culture in either the FBS- or FEx-supplemented media implying little impact on cellular metabolism (Figure S5 C, D). The analyses of the metabolomics data imply that the secreted cellular metabolites content when switched to FEx from FBS remains largely unchanged.

The inclusion of xenogenic components such as FBS used in *Drosophila* cell culture media has been a continued practice over several decades. In this study we have outlined a set of culture conditions for adapting *Drosophila* cells to grow in a serum-free media supplemented with fly extract. The key to achieving this transition for S2R+ cells is the supplementation with FEx, whereas the transition to serum-free media for Kc167 cells can be achieved without the supplementation of FEx via the proprietary HyClone-CCM3 serum-free media, standard M3 media alone, or with FEx supplementation in M3 medium. More importantly, cells grown in the presence of FEx retain similar growth characteristics, are amenable to transfection and are potentially exposed to conditions that elicit a lower oxidative stress as compared to FBS-cultured cells. Moreover, culturing S2R+ cells in FEx media did not globally alter either the transcriptome or the metabolome. These culture conditions therefore arguably provide a more native physiology that is devoid of serum-associated mitogenic factors for *Drosophila* cell cultures. Cells adapted to FBS-free media also provides researchers with an additional tool to investigate cell biological processes in a more physiological context. Specifically, it opens up a new avenue for researchers to explore the consequences of using FEx from particular metabolic mutants or flies reared under specific nutrient conditions to culture S2R+ cells.

Toward continued refinement of serum-free cell culture, we aim to adapt and characterize several other extensively used *Drosophila* cells lines. Furthermore, the culture conditions described in this study provide an opportunity to establish the minimal constituent(s) of FEx that are sufficient to support cell growth and proliferation. A more defined *Drosophila* cell culture condition will also aid studies aimed at assessing metabolite dynamics from or into the culture media, which is currently not a trivial endeavor. These prospective investigations in combination with the data that will be generated from *Drosophila* cell biologists utilizing these serum-free conditions will potentially clarify the critical requirements for *Drosophila* cell culture.

At the DGRC, fly extract is produced in up to 1L batches. Laboratories can obtain fly extract in bulk to use for extensive long-term experiments or in small amounts for short-term transient procedures. Currently there are no regulatory guidelines for the import and export trading of insect extract such as the fly extract. Therefore, it is considerably easier to import and export insect extracts, as compared to FBS, which has mammalian origins. Finally, we projected that the utilization of FEx also has a significant economic benefit to any laboratory that utilize *Drosophila* cells (Table S6), especially when cells are cultured in M3 + 2.5% FEx. This cost benefit will be even more significant if laboratories generate their own fly extract.
